# Supplementary material for: Age and Density of Mated Females Affect Dispersal Strategies in Spider Mite Tetranychus ludeni Zacher
Source: Insects. 2024 May 27;15(6):387. doi: 10.3390/insects15060387 (PMC11203671; doi:10.3390/insects15060387)
Supplement: Supplementary file 1 [file insects-15-00387-s001.zip › insects-2980040-supplementary.pdf]

Table S1. Statistical results of models of the egg density and offspring survival over distance in *T. ludeni* females of different ages in Figure 3.

**Treatment**	***a***	**95% CL**	***b***	**95% CL**	$F_{(df)}$	***p***
Eggs/cm²						
1 day old	2.1863	1.8823 ~ 2.4904	-0.1552	-0.2077 ~ -0.1027	$34.49_{(1, 89)}$	<0.0001
3 day old	2.8982	2.6334 ~ 3.1630	-0.1448	-0.1825 ~ -0.1071	$57.86_{(1, 125)}$	<0.0001
6 day old	2.5620	2.3129 ~ 2.8111	-0.1147	-0.1503 ~ -0.0792	$40.69_{(1, 132)}$	<0.0001
9 day old	2.4715	2.2319 ~ 2.7111	-0.0948	-0.1272 ~ -0.0624	$33.66_{(1, 118)}$	<0.0001
12 day old	2.5224	2.2864 ~ 2.7583	-0.1289	-0.1611 ~ -0.0967	$62.52_{(1, 141)}$	<0.0001
Offspring survival (%)						
1 day old	-0.1964	-0.2764 ~ -0.1164	0.01652	0.0009 ~ 0.0322	$4.40_{(1, 84)}$	0.0388
3 day old	-0.2828	-0.3797 ~ -0.1860	0.01841	0.0044 ~ 0.0325	$6.72_{(1, 124)}$	0.0107
6 day old	-0.2942	-0.3761 ~ -0.2124	0.01332	0.0019 ~ 0.0247	$5.34_{(1, 128)}$	0.0224
9 day old	-0.2663	-0.3433 ~ -0.1894	0.01498	0.0037 ~ 0.0263	$6.95_{(1, 113)}$	0.0096
12 day old	-0.2137	-0.2691 ~ -0.1583	0.00923	0.0010 ~ 0.0175	$4.92_{(1, 134)}$	0.0283

A logistic regression model, $y = \exp(a + bx)$, was used for analysis.
